# Balancing the CD38 Expression on Effector and Target Cells in Daratumumab-Mediated NK Cell ADCC against Multiple Myeloma

**DOI:** 10.3390/cancers13123072

**Published:** 2021-06-20

**Authors:** Margaux Lejeune, Elodie Duray, Matthias Peipp, Béatrice Clémenceau, Frédéric Baron, Yves Beguin, Jo Caers

**Affiliations:** 1 Laboratory of Hematology, GIGA I3, Department of Hematology, University of Liège, 4000 Liège, Belgium; Margaux.Lejeune@uliege.be (M.L.); E.Duray@uliege.be (E.D.); f.baron@uliege.be (F.B.); yves.beguin@chuliege.be (Y.B.); 2 Section for Stem Cell Transplantation and Immunotherapy, Department of Medicine II, Schleswig-Holstein University Medical Center, 24105 Kiel, Germany; m.peipp@med2.uni-kiel.de; 3 INSERM, CRCINA, University of Nantes, 44000 Nantes, France; beatrice.clemenceau@univ-nantes.fr; 4 Department of Hematology, CHU de Liège, 4000 Liège, Belgium

**Keywords:** ADCC, CD38, NK-92 cells, multiple myeloma, cellular cytotoxicity assays

## Abstract

**Simple Summary:**

We tracked the cytotoxic potential of NK cells towards multiple myeloma cells in daratumumab-mediated antibody-dependent cellular cytotoxicity assays. These cytotoxicity levels could be directly correlated to the expression of the target antigen (CD38) and to the percentage of fratricide between effector cells. Increasing the expression of CD38 on target cells or neutralizing CD38 on effector cells changed the equilibrium between target and effector cell lysis and promoted multiple myeloma cell death. This study highlights the importance of a balanced CD38 expression on target and effector cells and attempts to alter this balance will affect the susceptibility of MM cells towards daratumumab-mediated cellular toxicity.

**Abstract:**

Multiple myeloma (MM) is an incurable cancer characterized by the proliferation and accumulation of monoclonal plasma cells in the bone marrow. The monoclonal anti-CD38 daratumumab has taken a central place in the different treatment regimens for newly diagnosed and relapsed, refractory myeloma. In this study, we correlated the NK cell-mediated antibody-dependent cellular cytotoxicity (ADCC) and potential fratricide induced by daratumumab with CD38-expression levels on both effector and target cells. We show that CD38 expression can be modulated by adding all-trans retinoic acid (ATRA) or interferon-α to MM cells to further fine-tune these effects. In addition, we observed that ADCC becomes inefficient when fratricide occurs and both ADCC and fratricide depend on the balance between CD38 expression on effector and target cells. However, the addition of adjuvants (retinoic acid or interferon-α) to myeloma cells or the inhibition of fratricide using a CD38-blocking nanobody on NK-cells can reverse this balance towards ADCC and thus promote lysis of target cells by ADCC. ATRA and interferon-α increased the CD38 expression at the surface of MM cells about three-fold and two-fold, respectively. This increase was of interest for MM cells with low CD38 expression, that became susceptible to daratumumab-mediated ADCC after preincubation. A CD38-blocking nanobody prevented the binding of daratumumab to these NK-cells and blunted the fratricidal effect on effector NK cells. In conclusion, our study highlights the importance of a balanced CD38 expression on target and effector cells and attempts to alter this balance will affect the susceptibility of MM cells towards daratumumab-mediated ADCC.

## 1. Introduction

The survival of patients with multiple myeloma (MM) has dramatically improved with the introduction of autologous stem cell transplantation and new drug classes such as proteasome inhibitors and immunomodulatory drugs. Despite this progress, the vast majority of patients relapse, underscoring the need for new treatment options. In recent years, different forms of immunotherapy have been developed to tackle more selectively MM cells and to obtain long-lasting responses with fewer side effects. The introduction of antibody-based treatments such as monoclonal antibodies (mAbs) or bispecific antibodies tries to answer this unmet clinical need [1,2].

With introduction of mAbs, researchers focused on the activity of Natural Killer (NK) cells in MM, as they represent one of the major effectors, with macrophages, of mAbs. NK cells are granular cytotoxic lymphocytes of the innate immune system that can target malignant cells without prior sensitization. Among other mechanisms, antibodies induce cell death by recruiting NK-cells for an antibody-dependent cellular cytotoxicity (ADCC) [3,4].

Daratumumab, an anti-CD38 human IgG1, is the most promising mAb for the treatment of MM [5]. CD38 is a type II transmembrane glycoprotein that is highly and uniformly expressed on malignant plasma cells at all stages of the disease [6]. This antigen is also moderately expressed by mature lymphocytes and non-hematopoietic tissues. Daratumumab has a limited toxicity profile, while exhibiting activity as monotherapy and allowing therapeutic combinations with existing therapies [7]. It was approved for Relapsed/Refractory Multiple Myeloma (r/r MM) by the FDA in 2015 [8] and by the EMA in 2016 based on the results of the GEN501 and SIRIUS clinical studies which demonstrated that daratumumab has promising anti-myeloma activity [9,10]. Both regulatory organizations approved the association of daratumumab with either bortezomib or lenalidomide for r/r MM, based on the impressive improvements in response rates and progression free survival [11,12,13].

However, treatment with daratumumab causes rapid depletion of around 85% of NK cells, lasting from 3 to 6 months after treatment discontinuation [14,15]. This NK cell depletion is attributed to their CD38-expression. The use of NK cells weakly expressing CD38 could thus be a strategy to optimize the efficacy of daratumumab [16]. In the past different sources of NK cells have been used, ranging from NK cell lines, expanded primary NK cells or stem-cell derived and differentiated NK-cells. The best-known NK cell line is the NK-92 cell line, which is derived from a patient with Hodgkin’s lymphoma. Due to its malignant nature, this NK-92 cell line must be irradiated before infusion into the patient. Even after irradiation, it has a strong cytotoxic potential and NK-92 infusions are safe and well tolerated even at high doses [17]. Clinical studies have already been carried out by injecting the irradiated NK-92 cells and anti-tumor effects could be observed without persistence of NK-92 cells after treatment [18,19].

This study investigates the ADCC-dependent cytotoxic effects of daratumumab according to CD38 expression levels on the different cells and the impact of modulating CD38 expression in target and effector cells. We integrate two different methods to quantify ADCC (one based on flow cytometry and one based on calcein release assay) in order to better distinguish the effects on both target and effector populations.

## 2. Materials and Methods

### 2.1. Cell Lines

LP-1 cells were cultured in Dulbecco’s Modified Eagle’s Medium (DMEM) (Lonza, Verviers, Belgium) supplemented with 10% fetal bovine serum (FBS; Sigma-Aldrich, St-Louis, MO, USA), 2 mM L-glutamine (Lonza, Verviers, Belgium) and 100 U/mL penicillin-streptomycin (P/S; Lonza, Verviers, Belgium). RPMI-8226 cells, U266 cells, MOLP-2 cells and K562 cells were cultured in Roswell Park Memorial Institute (RPMI 1640) (Lonza, Verviers, Belgium) supplemented with 10% fetal bovine serum (FBS; Sigma-Aldrich, St-Louis, MO, USA), 2 mM L-glutamine (Lonza, Verviers, Belgium) and 100 U/mL penicillin-streptomycin (P/S; Lonza, Verviers, Belgium). NK-92 cells and NK-92 hCD16a cells were cultured in RPMI Medium 1640 + GlutaMAX^TM^-I (Lonza, Verviers, Belgium) supplemented with 10% fetal bovine serum (FBS; Sigma-Aldrich, St-Louis, MO, USA), 25 mM HEPES, 100 U/mL penicillin-streptomycin (P/S; Lonza, Verviers, Belgium) and 5 ng/mL d’IL-2 (Peprotech, Neuilly-sur-Seine, France). NK-92 hCD16a cells were provided by Béatrice Clémenceau (Centre de Recherche en Cancérologie et Immunologie Nantes-Angers (CRCINA), Université d’Angers-Nantes, France) [20]. All cell lines were cultured at 37 °C in 5% CO_2_ humidity.

### 2.2. CRISPR Plasmids

As specific controls, we generated LP-1 and RPMI-8226 CD38-knock out (KO) cell lines. We used the CRISPR/Cas9 technology (Santa Cruz Biotechnology, Dallas, TX, USA). Lentiviral plasmids containing guide RNA targeting the human CD38 gene (on chromosome 4) for the CRISPR system were purchased from Sigma-Aldrich (St-Louis, MO, USA) (HSPD0000006112 (Target Sequence: TTGACGCATCGCGCCAGGACGG), HSPD0000006113 (Target Sequence: CACCGCGAGCACCACGACGAGG) and HSPD0000006114 (Target Sequence: CTGGAAAACGGTTTCCCGCAGG): U6-gRNA:hPGK-puro-2A-tBFP). This allows expression of guide RNA (under U6 promoter) and includes a puromycin resistance gene fused to turbo BFP sequence under human PGK promoter. Another lentiviral plasmid was used for high specific SpCas9 expression [21]. This was purchased at VectorBuilder (Neu-Isenburg, Germany (VB190913-1028vgu): pLV LoxP-EF1A espCas9(ns)-T2A-mCherry-LoxP. This plasmid allows expression of espCAS9 as well as mCherry.

### 2.3. Lentiviral Vector Production and Cell Transduction

Lenti-X 293T cells (Clontech^®^, Takara Bio Europe SAS, Saint-Germain-en-Laye, France) were co-transfected together with lentiviral gene transfer plasmids (U6-gRNA:hPGK-puro-2A-tBFP or pLV LoxP-EF1A espCas9(ns)-T2A-mCherry-LoxP) and packaging plasmids (pSPAX2 (Addgene plasmid # 12260) and a VSV-G encoding plasmids) [22]. Lentiviral supernatants were collected 48 h, 72 h and 96 h post transfection, filtrated and concentrated 100× by ultracentrifugation. Lentiviral vectors were then titrated with qPCR Lentivirus Titration (Titer) Kit (ABM^®^, LV900, Richmond, BC, Canada) and used to transduce LP-1 cells.

Cells were double transduced with CRISPR lentiviral vectors and lentiviral guide RNA for CD38 using 30 lentiviral particles per cells. After 72 h, cells expressing mCherry and BFP were isolated by FACS (BD Biosciences, San Jose, CA, USA) and then maintained in culture. Cells that were not expressing CD38 receptor anymore were selected by FACS using the APC-conjugated CD38 mAb (HIT2, BioLegend, San Diego, CA, USA). This step was done twice and the KO for CD38 was confirmed by Western blot.

### 2.4. Staining by Flow Cytometry

Membrane stainings were performed on different cell lines (LP-1, RPMI-8226, U266, MOLP-2, NK-92, NK-92 CD16a) and primary NK cells. Cells were incubated for 30 min at 4 °C in the presence of predefined antibody concentrations before analysis by flow cytometry. The following antibodies were used: Human TruStain FcX^TM^ (BioLegend, San Diego, CA, USA), CD38-PE (HIT2, BD Biosciences, San Jose, CA, USA), CD38-APC (HIT2, BioLegend, San Diego, CA, USA). Flow cytometry analyses were performed on a FACSCanto II flow cytometer (BD Biosciences, San Jose, CA, USA) and data were analyzed using BD FACSDiva Software (BD Biosciences, San Jose, CA, USA).

### 2.5. Quantification of CD38 Expression

LP-1, RPMI-8226, U266, MOLP-2 and NK-92 cell lines and primary NK cells were labelled with a PE-conjugated monoclonal antibody and analyzed by FACS. Non-specific binding was blocked with Fc-gamma receptor antibodies. We used the BD Quantibrite™ Beads (BD Biosciences, San Jose, CA, USA) to quantify the CD38 expression according to the manufacturer’s instructions. This kit contains a tube with beads conjugated at four levels of PE intensity. These 4 intensity levels are associated with a specific number of PE molecules per bead. Based on the generated standard curve, we were able to calculate the number of PE molecules per cell after labeling the cells with a PE-labeled mAb.

### 2.6. Irradiation of Effector Cells

Effector cells (NK-92 and NK-92 CD16a) were irradiated at 10 or 20 Gy using a ^137^Cs source (GammaCell 40, Nordion, Ontario, ON, Canada) and washed with complete medium after irradiation. The cells were then co-cultured with target cells to assess their efficacy after irradiation according to methods described below.

### 2.7. PBMCs and NK Cells Isolation

Human whole peripheral blood (PB) was obtained from healthy adult volunteers following written informed consent. PBMCs were isolated by Ficoll-Paque density centrifugation (GE Healthcare, Freiburg, Germany). Primary NK cells were purified using the EasySep Human NK Cell Isolation Kit (Stemcell Technologies, Vancouver, YVR, Canada) according to the manufacturer’s protocol. The cells were then co-cultured with target cells to assess their efficacy according to methods described below.

### 2.8. Incubation of Target Cells with Adjuvants

To increase the expression of CD38 on the surface of the target cells, LP-1, RPMI-8226, U266 and MOLP-2 cells, they were incubated in the presence of all-trans retinoic acid (ATRA; Sigma-Aldrich, Saint-Louis, MO, USA) and interferon α (IFNα; Sigma-Aldrich, Saint-Louis, MO, USA). A concentration of 0.5 μM and 2500 pg/mL of ATRA and IFNα, respectively, were added to the cells in culture. These cells were then incubated for 3 days at 37 °C in 5% CO_2_ humidity before being used in the experiments.

### 2.9. Incubation of Effector Cells with Anti-CD38 Nanobody or Anti-NKG2A Antibody

NK-92 CD16a effector cells were washed twice in PBS with 3% FBS. They were then incubated in the presence of 5 μg of anti-CD38 nanobody (Nb551; provided by Zhao, Peking University Shenzhen Graduate School, Shenzhen, China) [23] or of 5 μL of anti-NKG2A antibody (CD159a; Z199, Beckman Coulter, Brea, CA, USA) at 4 °C for 30 min. They were again washed twice before being used in the experiments.

### 2.10. Cellular Cytotoxicity Assays: Flow Cytometry

NK-92—dependent tumor cell cytotoxicity was quantified by flow cytometry. Target cells were labeled using CellTrace™ Violet (Thermo Fisher Scientific, Waltham, MA, USA) and were incubated for 20 min at 37 °C in humidified atmosphere with 5% CO_2_ at an effector-to-target ratio of 10:1. After washing, target cells were re-suspended in supplemented media and mixed in co-culture with effector cells in the presence of 10 µg/mL of Daratumumab [24] (Janssen Pharmaceuticals, Spring House, PA, USA) in 96-well plates for 6 h at 37 °C in a humidified atmosphere with 5% CO_2_. After 6 h, dead violet dye-labeled tumor cells were measured with 7-AAD Viability Staining Solution (Thermo Fisher Scientific, Waltham, MA, USA) by flow cytometry 5 min after its addition. Specific cytotoxicity was determined by the equation: % dead tumor cells − % spontaneous tumor cell death. The leukemia cell line K562 was used as a negative control for NK-92—dependent cytotoxicity. The gating strategy is described in Figure S1.

### 2.11. Cellular Cytotoxicity Assays: Calcein Release Assay

NK-92—dependent tumor cell cytotoxicity was also measured by the calcein release assay. Target MM cells were incubated with 5 µM of calcein-AM for 30 min. After washing, target cells were re-suspended in supplemented media and mixed with effector cells in the presence of 10 µg/mL of daratumumab in 96-well plates for 2 h or 4 h at 37 °C in a humidified atmosphere with 5% CO_2_ at the indicated effector-to-target ratio. Fluorescence was measured in the supernatants of each well using a fluorescence plate reader. Each condition was performed in triplicate. The percentage of specific lysis was calculated using the equation (Fluorescence (sample) − Fluorescence (spontaneous))/(Fluorescence (maximum) − Fluorescence (spontaneous)) × 100% and expressed as the mean of triplicate samples. The LP-1 and RPMI-8226 CD38-KO cells were used as negative controls for NK-92—dependent cytotoxicity.

### 2.12. Statistical Analyses

Results are shown as means ± standard error and representative pictures are shown. For comparisons of 2 mean values, an unpaired *t*-test was used. All statistical analyses were performed with Prism 5 software (GraphPad software, San Diego, CA, USA). *p*-values below 0.05 were considered significant and represented as follows: * *p* < 0.05, ** *p* < 0.01, *** *p* < 0.001, **** *p* < 0.0001. *p*-values above 0.05 were considered non-significant and are represented as “ns”.

## 3. Results

### 3.1. ADCC—Dependent Tumor Cell Cytotoxicity: Flow Cytometry

**Figure 1:**
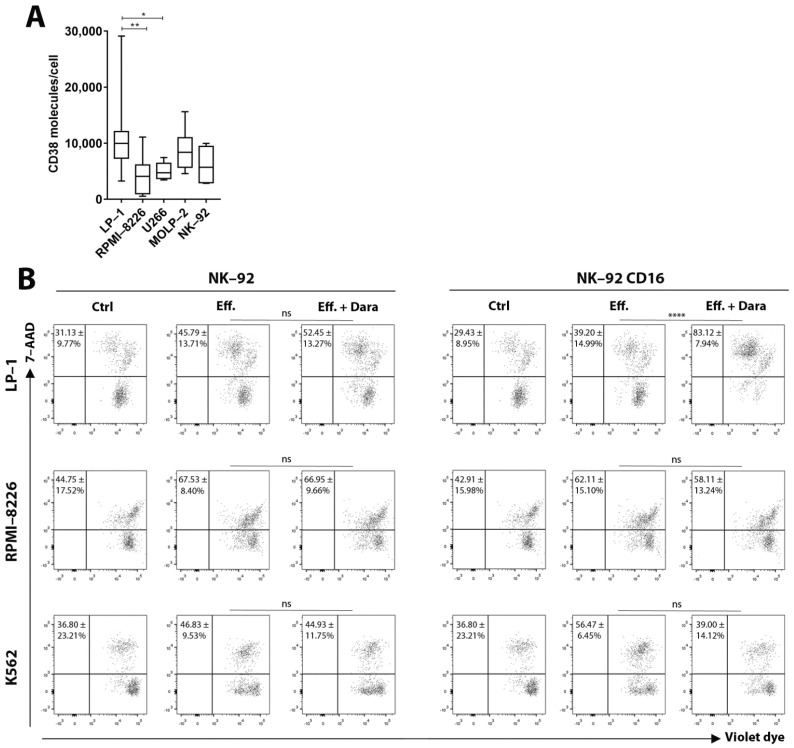
NK-92—dependent tumor cell cytotoxicity after 6 h of co-culture and measured by flow cytometry. (**A**) Quantification of number of CD38 molecules on the surface of LP-1, RPMI-8226, MOLP-2, U266 and NK-92 cells. Results are representative of six independent experiments (*n* = 6). (**B**) Dot plots with percentages of cell apoptosis after 6 h of incubation in absence of effector cells (Ctrl) or in presence of NK-92 or NK-92 CD16a cells in an effector-to-target cells ratio of 10:1, with (Eff. + Dara) or without (Eff.) daratumumab. Numbers in quadrants represent average percentage ± standard deviation of gated cells. Plots show violet dye versus 7-AAD fluorescence. Experiments were realized at least in triplicate. Ctrl: control. Eff: effector cells. Dara: daratumumab. ns: non-significant. *: *p* < 0.05. **: *p* < 0.01. ****: *p* < 0.0001.

We first quantified CD38 expression on the surface of MM cells (target cells) and NK-92 (effector cells) (Figure 1A). To allow their application in ADCC assays, the NK-92 cell line, transduced with the human FcγRIIIa (CD16a) receptor and termed hereafter NK-92 CD16a, was used [20]. Flow cytometry confirmed daratumumab-induced cell lysis of LP-1 cells after 6 h of co-culture with NK-92 CD16a effector cells. In contrast, no lysis was seen in RPMI-8226 cells (Figure 1B). The parental NK-92 cells did not induce ADCC. We observed a 44% increase of NK-92 CD16a cell cytotoxicity towards LP-1 cells in the presence of daratumumab. No increase in cytotoxicity was seen when the CD38-negative K562 cell line was used as a negative control.

The different ADCC results could potentially be explained by the CD38 expression on the surface of target cells. Quantification of the number of surface CD38 molecules showed that CD38 expression was 2.5 times higher on LP-1 cells (±10,800 CD38/cell) than on RPMI-8226 cells (±4400 CD38/cell) (Figure 1A). Because basal cytotoxicity levels (without daratumumab) of NK-92 CD16a towards RPMI-8226 cell was already high (Figure 1B), small differences in ADCC could be missed by this assay.

Irradiation of NK-92 and NK-92 CD16a cells with doses of 10 Gy blocked their proliferation but did not affect the cytotoxic activity of the two cell lines (Figure S2). Increasing the radiotherapy dose up to 20 Gy decreased their cytotoxic capacities. We concluded that an irradiation dose of 10 Gy is sufficient to maintain the cytotoxic activity of NK-92 CD16a cells towards the LP-1 and RPMI-8226 cell lines and to stop their proliferation.

### 3.2. Fratricide between NK-92 CD16a Effector Cells: Flow Cytometry

**Figure 2:**
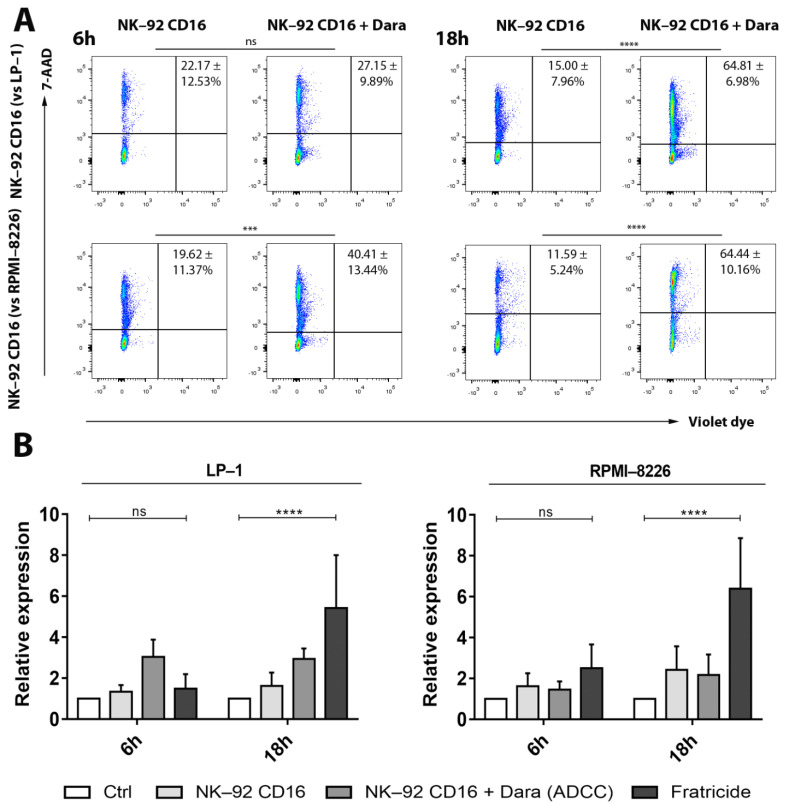
Fratricide between NK-92 CD16a effector cells after 6 h of co-culture and measured by flow cytometry. (**A**) Representative dot plots of fratricide percentages between NK-92 CD16a cells after 6 h and 18 h of incubation in presence of target cells in an effector-to-target cells ratio of 10:1. Numbers in quadrants represent average percentage ± standard deviation of gated cells. The phenomenon of fratricide only occurs in presence of daratumumab. Plots show violet dye versus 7-AAD fluorescence. (**B**) Relative expression of ADCC (NK-92 CD16 + Dara) and fratricide rates after 6 h and 18 h of co-culture (LP-1 or RPMI-8226 target cells with NK-92 CD16a effector cells) in an effector-to-target cells ratio of 10:1. All figures are representative of at least 3 independent experiments and represented as mean ± standard error. Ctrl: control. Dara: daratumumab. ns: non-significant. ***: *p* < 0.001. ****: *p* < 0.0001.

In addition to the anti-tumoral effects, we retrieved the previously described fratricide phenomenon between NK-92 CD16a cells in the presence of daratumumab. This fratricide increases over time during co-cultures with target cells: after 6 h and 18 h of incubation with daratumumab and LP-1 cells, 27% and 65% of NK-92 CD16a cells are lysed, respectively; incubation with RPMI-8226 induced 40% and 65% of NK-92 CD16a cell lysis at the same time points (Figure 2A). On the other hand, no fratricide was observed between wild-type NK-92 cells in the presence of daratumumab (data not shown), highlighting the fact that the phenomenon of fratricide occurs via the ADCC mechanism. Interestingly, these levels of fratricide depend on the target cell lines used and were higher during co-culture with RPMI-8226 cells (40%) after 6 h of incubation compared to co-culture with LP-1 cells (27%) (Figure 2A).

This is also potentially explained by levels of CD38 expression on the surface of both target and effector cells. Quantification of the number of CD38 molecules indicated a lower expression on the surface of NK-92 cells (±6000 CD38/cell) compared to LP-1 cells (±10,800 CD38/cell), but higher compared to RPMI-8226 cells (±4400 CD38/cell) (Figure 1A). The level of fratricide is correlated with ADCC levels and both are dependent on CD38-expression on target and effector cells. When we directly compared the results obtained by ADCC to those obtained on fratricide, we observed, that ADCC of the LP-1 cell line after 6 h of co-culture was almost twice as high as fratricide, but this balance was reversed after 18 h of co-culture (Figure 2B).

On the other hand, for the RPMI-8226 cell line, fratricide was almost twice as high as ADCC after 6 h of incubation and three times higher after 18 h of incubation (Figure 2B).

### 3.3. Confirmation of Cytotoxicity and Fratricide by the Calcein Release Assay

The flow cytometry protocol only allows quantification of cell death at a specific timepoint. The calcein release assay, with the advantage of avoiding radioactive labeling compared to the chromium release assay, allows to monitor the accumulation of cytotoxicity. In addition, basal cytotoxicity towards the LP-1 and RPMI-8226 cell lines is already very high at the effector/target ratio of 10:1 (40% and 62%, respectively) (Figure 1B) making it more difficult to visualize small differences in ADCC. Therefore, we tested lower effector-to-target ratios (1:1, 0.5:1, 0.25:1) in order to decrease basal cytotoxicity. Furthermore, we reduced incubation time from 6 h to 2 and 4 h of incubation to minimize natural cytotoxicity.

**Figure 3:**
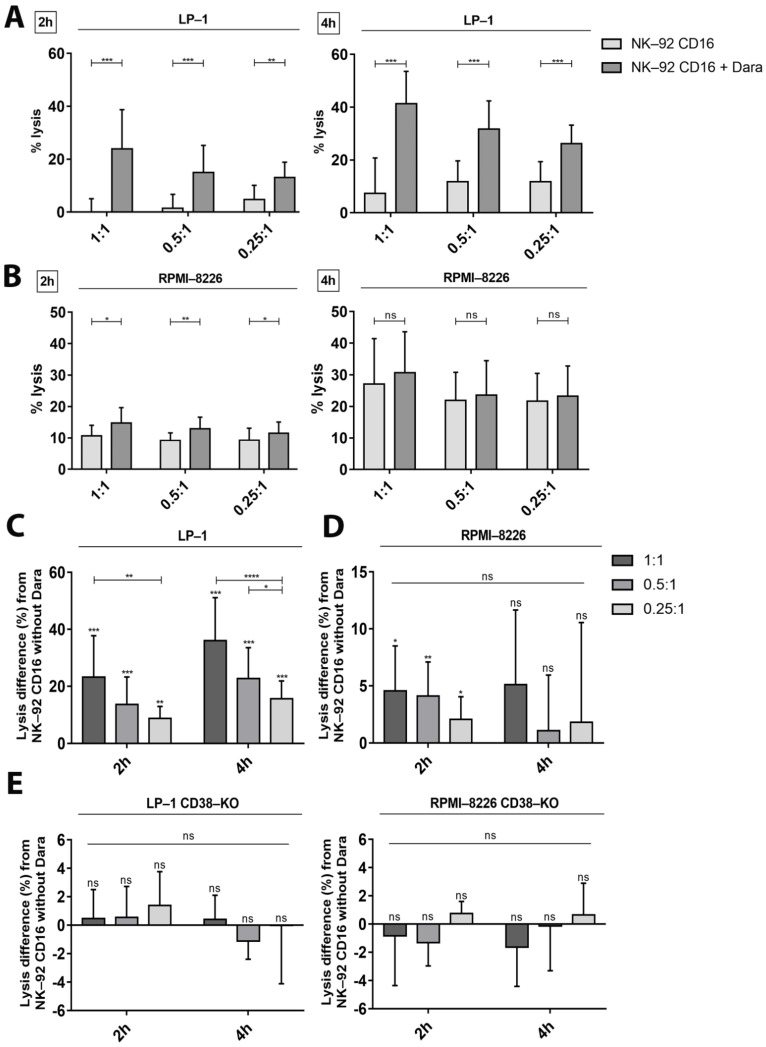
NK-92—dependent tumor cell cytotoxicity and fratricide measured by calcein release assay. Lysis percentages of LP-1 (**A**) and RPMI-8226 (**B**) after 2 h and 4 h of incubation in presence of effector cells with (NK-92 CD16 + Dara) or without (NK-92 CD16) daratumumab. The effector-to-target cells ratios tested were 1:1, 0.5:1 or 0.25:1. Lysis differences in percentage of LP-1 (**C**), RPMI-8226 (**D**) and CD38-KO cell lines (LP-1 and RPMI-8226) (**E**) after 2 h or 4 of incubation between conditions in presence of NK-92 CD16a with or without daratumumab, in different E/T ratios (1:1, 0.5:1 or 0.25:1). All data are representative of at least 3 independent experiments and represented as mean ± standard error. Dara: daratumumab. ns: non-significant. *: *p* < 0.05. **: *p* < 0.01. ***: *p* < 0.001. ****: *p* < 0.0001.

As expected, decreasing E/T ratios and incubation time limited basal cytotoxicity towards the LP-1 and RPMI-8226 cell lines. LP-1 cell lysis rates were 0%, 1.4% and 4.7% at the three E/T ratios, respectively, after 2 h of incubation, and 7.3%, 11.8% and 11.8%, respectively, after 4 h (Figure 3A). For RPMI-8226 cells, basal lysis was 10.7%, 9.2% and 9.3% after 2 h and 27.0%, 21.9% and 21.7% after 4 h, respectively (Figure 3B).

The calcein release assay confirmed ADCC towards LP-1 cells (Figure 3C). Cytotoxicity appeared already after 2 h of incubation. Cytotoxicity of NK-92 CD16a cells towards LP-1 cells in the presence of daratumumab increased by 23.2%, 13.6% and 8.7%, respectively, after 2 h of incubation and by 36.0%, 22.7% and 15.6%, respectively, after 4 h.

On the other hand, low levels of ADCC towards RPMI-8226 cells were observed after 2 h of incubation (Figure 3D). We observed increases of 4.6%, 4.1% and 2.0%, respectively, in the cytotoxicity of NK-92 CD16a cells towards RPMI-8226 cells in the presence of daratumumab after 2 h of incubation. We no longer observed ADCC lysis after 4 h of incubation (Figure 3D). This observation could be explained by the high basal cytotoxicity of NK-92 CD16a cells towards these RPMI-8226 cells (Figure 3B), which masks potential increases with daratumumab.

Finally, we also tested the two CD38-KO cell lines (LP-1 and RPMI-8226) generated by the CRISPR/Cas9 technology (Figure S3). We did not observe any ADCC in the presence of daratumumab towards the CD38-KO LP-1 and RPMI-8226 cell lines after 2 h and 4 h of incubation (Figure 3E).

**Figure 4:**
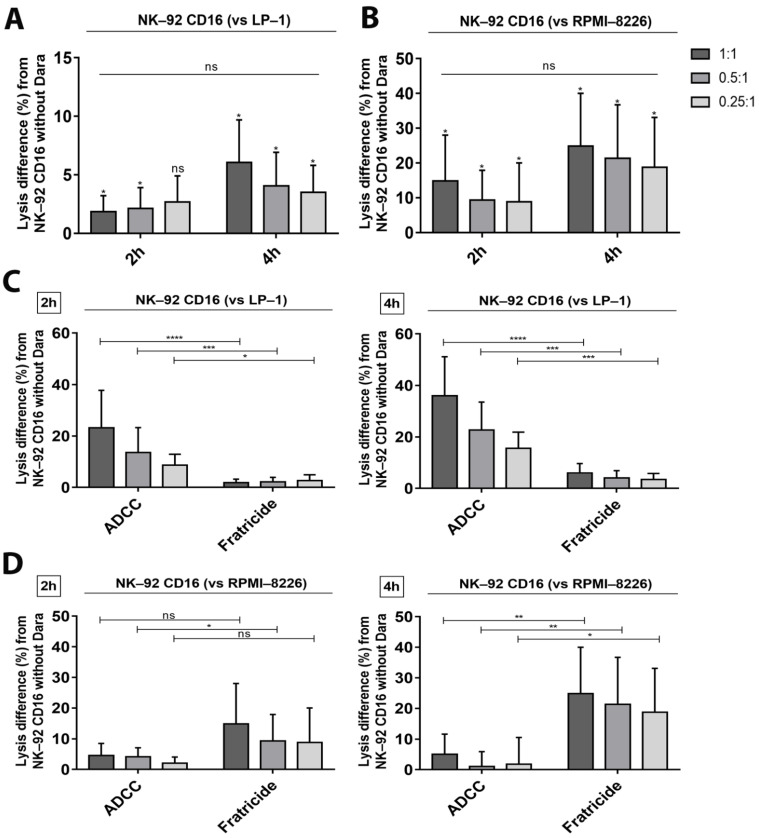
Fratricide between NK-92 CD16a effector cells measured by calcein release assay. Fratricide percentages differences between NK-92 CD16a cells with or without daratumumab after 2 h and 4 h of co-culture with target cells LP-1 (**A**) or RPMI-8226 (**B**) in effector-to-target cells ratios of 1:1, 0.5:1 and 0.25:1. Comparison of percentages of ADCC and fratricide in co-cultures with LP-1/NK-92 CD16a (**C**) or RPMI-8226/NK-92 CD16a co-cultures (**D**). All data are representative of six independent experiments and represented as mean ± standard error. Dara: daratumumab. ns: non-significant. *: *p* < 0.05. **: *p* < 0.01. ***: *p* < 0.001. ****: *p* < 0.0001.

The calcein release assay also allows tracking the daratumumab-induced fratricide phenomenon between NK-92 CD16a cells during co-cultures with target cells. Indeed, fratricide increased with time and depended on the target cell line. Fratricide was higher during co-culture with RPMI-8226 cells than with LP-1 cells (14.8% vs. 1.9% and 24.8% vs. 6.1%, after 2 and 4 h of co-culture, respectively, at 1:1 ratio) (Figure 4A,B).

**Figure 5:**
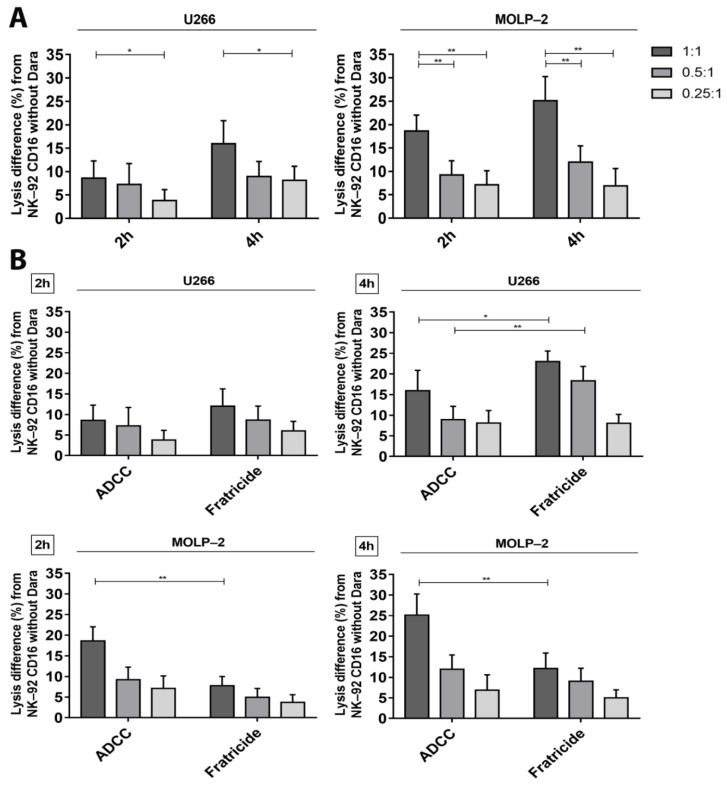
ADCC and fratricide results measured by calcein release assay with U266 and MOLP-2 cells. (**A**) Lysis differences in percentage of U266 and MOLP-2 cells by NK-92 CD16a cells after 4 h of incubation. (**B**) Fratricide percentages differences between NK-92 CD16a with or without after 2 and 4 h of co-cultures with U266 or MOLP-2 cells. All experiments compare conditions in presence of NK-92 CD16a with or without daratumumab and, in different E/T ratios (1:1, 0.5:1 or 0.25:1). All data are representative of five (*n* = 5) independent experiments and represented as mean ± standard error. *: *p* < 0.05. **: *p* < 0.01.

To confirm direct correlation between CD38 expression, ADCC and fratricide, we tested two additional MM cell lines, U266 and MOLP-2, who present an intermediate CD38 expression (±5000 and ±8800 molecules of CD38 per cell, respectively) (Figure 1A). ADCC assays showed cytotoxic effects proportional to their CD38 expression. Indeed, lysis of U266 cells is lower than lysis of MOLP-2 (Figure 5A), but higher than lysis of RPMI-8226 cells. The degree of MOLP-2 cells lysis by ADCC was intermediate between LP-1 and U266 cells, in accordance with their respective CD38 expression (Figure 5A). Fratricide of NK-92 CD16a was low in co-cultures with MOLP-2 cells but increased in co-cultures with U266 cells where it was 1.4 times higher than ADCC after 2 h of incubation (Figure 5B). However, fratricide did not hamper U266 cell lysis, although this remained low.

### 3.4. Increase of CD38 Expression after Adding of Adjuvants

To verify that modifications in CD38 expression affect the ADCC results, we increased CD38 expression on target cells by adding ATRA and IFNα.

**Figure 6:**
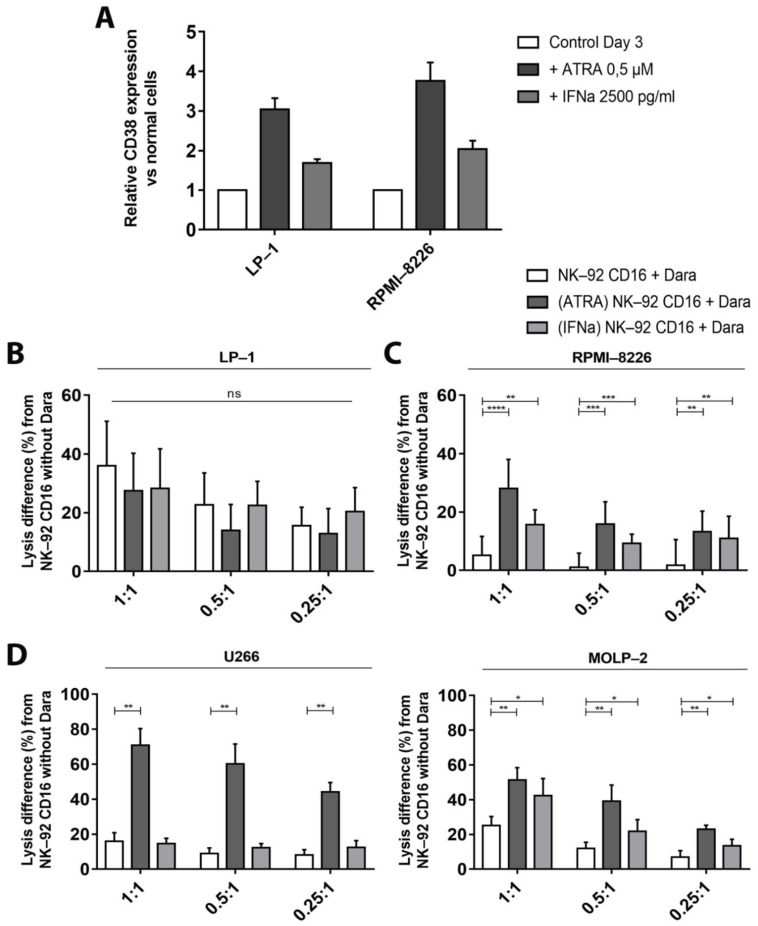
The effect of increasing CD38 density on NK-92—dependent tumor cell cytotoxicity, measured by calcein release assay. Results are representative of three independent experiments (*n* = 3). (**A**) Relative expressions of CD38 on the surface of LP-1 and RPMI-8226 target cells after 3 days of incubation with 0.5 µM of ATRA or with 2500 pg/mL of IFNα compared to cells alone. Lysis differences in percentage of LP-1 (**B**), RPMI-8226 (**C**), U266 or MOLP-2 (**D**) pre-incubated with ATRA or IFNα for 3 days, after 4 h co-cultures with or without daratumumab, in different E/T ratios (1:1, 0.5:1 or 0.25:1). Experiments were realized at least in triplicate and represented as mean ± standard error. Dara: daratumumab. ns: non-significant. *: *p* < 0.05. **: *p* < 0.01. ***: *p* < 0.001. ****: *p* < 0.0001.

We first followed CD38 expression on the two MM cell lines, LP-1 and RPMI-8226, in the presence of different concentrations of ATRA and IFNα (data not shown). Based on these results, we retained concentrations of 0.5 μM of ATRA and 2500 pg/mL of IFNα which increased the CD38 levels by 3 or 2 times, respectively (Figure 6A).

Cytotoxicity tests were performed with pre-incubated target cells. Although CD38 expression increased, we did not observe any increase in LP-1 cell lysis after adding ATRA or with IFNα. (Figure 6B).

On the other hand, ADCC-mediated cytotoxicity towards RPMI-8226 cells significantly increased after pre-incubation (Figure 6C). Indeed, increases up to 23.0% of NK-92 CD16a-mediated cytotoxicity towards RPMI-8226 cells were seen after pre-incubation with ATRA compared to cells not pre-incubated with ATRA. Similarly, pre-incubation with IFNα increased the percentages of RPMI-8226 cytotoxicity up to 10.5% compared to control cells. These percentages are higher with ATRA compared to IFNα, probably explained by the higher CD38 levels obtained with ATRA (Figure 6A). Pre-incubation with these adjuvants sufficiently increased the CD38 expression to reverse the balance between ADCC and fratricide and favor ADCC. We also incubated the effector cells with the adjuvants and put them in co-culture with the target cells pre-incubated or not with the adjuvants. While pre-incubation of target cells only with ATRA or IFNα promotes ADCC. In contrast, CD38-upregulation on effector cells promoted fratricide of NK-92 CD16a cells. The relative increase in CD38 expression was higher on NK-92a cells compared to myeloma cells when both populations were preincubated with ATRA or IFNα. We observed an increase in fratricide, that correlated well with the upregulation of CD38-expression on NK-92 CD16a cells. These results support our initial hypothesis that the balance between ADCC and fratricide depends on the CD38-expression, because ATRA and IFNα induced a higher increase on NK-92 CD16a cells compared to LP-1 and RPMI-8226 cells (Figure S4).

The effects of ATRA on CD38 expression and subsequent ADCC were confirmed on U266 and MOLP-2 cell lines (Figure 6D). This increase was markedly stronger for U266 cells than for MOLP-2 cells. The observed differences were in line with results obtained with LP-1 and RPMI-8226 cells according to their respective basal CD38 expression. Pre-incubation with IFNα did not significantly increase ADCC towards U266 or MOLP-2 cells (Figure 6D).

Fratricide inhibition between NK-92 CD16a cells with a blocking anti-CD38 nanobody.

**Figure 7:**
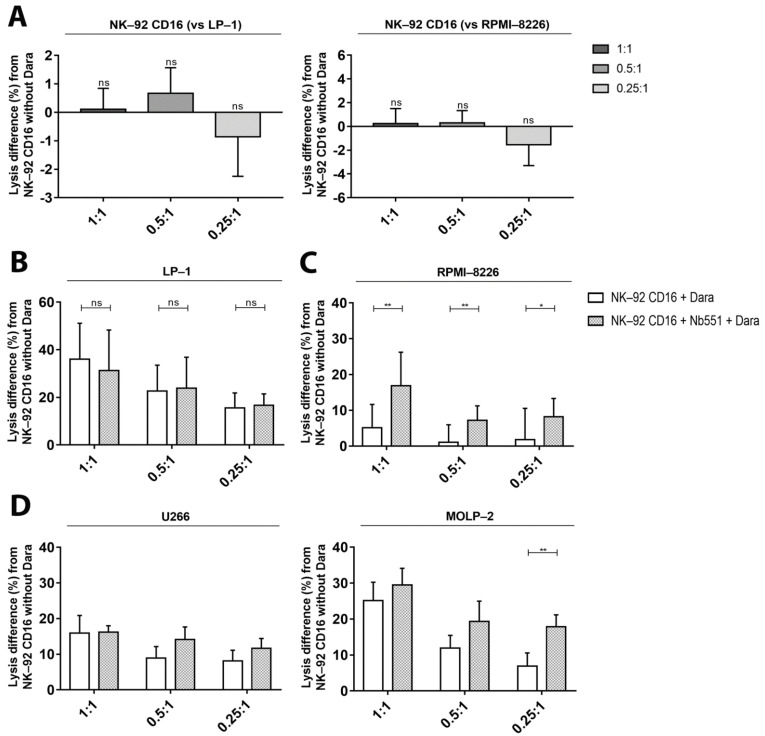
The effect of a blocking anti-CD38 nanobody (Nb551) on NK-92—dependent tumor cell cytotoxicity measured by calcein release assay (**A**) Differences in fratricide percentages differences between NK-92 CD16a cells without daratumumab, with daratumumab and with daratumumab and Nb551 in co-cultures with LP-1 or RPMI-8226 target cells (effector-to-target cells ratios of 1:1, 0.5:1 and 0.25:1). All data are representative of five (*n* = 5) independent experiments and represented as mean ± standard error. Target-cell lysis of LP-1 (**B**) and RPMI-8226 (**C**), U266 or MOLP-2 (**D**) after 4 h of co-culturing with NK-92 CD16a with or without daratumumab (E/T ratios of 1:1, 0.5:1 or 0.25:1). All data are representative of at least 3 independent experiments and represented as mean ± standard error. Dara: daratumumab. ns: non-significant. *: *p* < 0.05. **: *p* < 0.01.

To further investigate the effects of blocking CD38 binding on effectors cells, we used a competitive nanobody (Nb551) that targets the same epitope on CD38 as daratumumab. Blocking this epitope hampered the binding of daratumumab to CD38 on effector cells and inhibited the fratricide between NK-92 CD16a cells (Figure 7A).

Pre-incubation of NK-92 CD16a effector cells with 5 μg of Nb551 for 30 min at 4 °C had no effects on daratumumab-mediated cytotoxicity towards LP-1 cells (Figure 7B) but increased cytotoxicity up to 12% towards RPMI-8226 cells compared to NK-92 CD16a without pre-incubation (Figure 7C). As with the effects with ATRA and IFNα, inhibition of fratricide increased the ADCC activity of NK-92 CD16a in co-cultures with RPMI-8226 cells. On the other hand, pre-incubation of target cells with Nb551 prevented binding of daratumumab to myeloma cells and subsequent ADCC activity. This inhibition resulted in an increase in fratricide (Figure S5).

When competitive nanobody was used in ADCC assays with U266 and MOLP-2 cells, we observed minor differences in cytotoxicity (Figure 7D).

### 3.5. ADCC—Dependent Tumor Cell Cytotoxicity after Increase of CD38 Expression and Fratricide Inhibition

**Figure 8:**
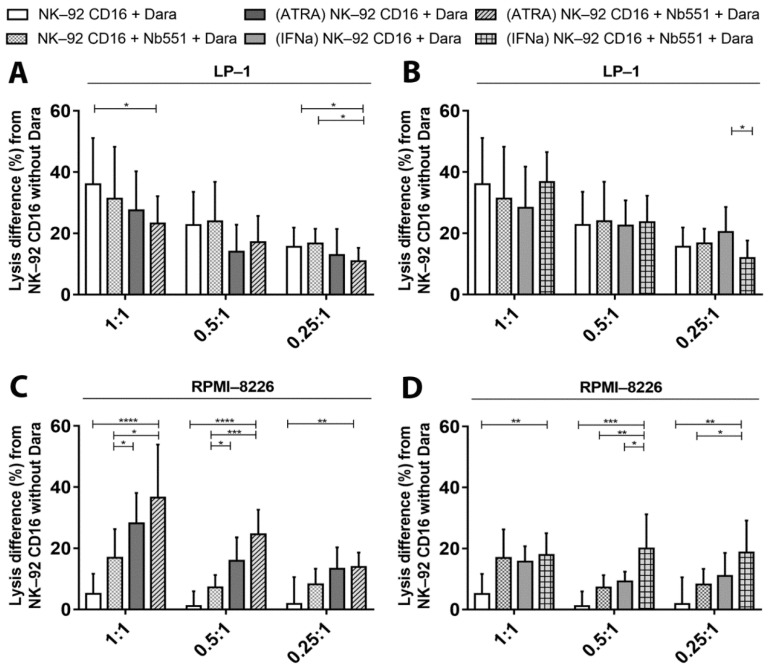
Effect on NK-92 cytotoxicity by combining adjuvants to increase in CD38 density on target cells and blocking nanobodies for CD38 on effector cells. (**A**) Differences in LP-1 cell lysis, pre-incubated or not with ATRA for 3 days, by NK-92 CD16a cells that were on their hand pre-incubated or not with Nb511. (**B**) Instead of ATRA, preincubation of LP-1 cells was done with IFNα for 3 days (**C**) Differences in RPMI-8226cell lysis, pre-incubated or not with ATRA for 3 days, by NK-92 CD16a cells that were on their hand pre-incubated or not with Nb511. (**D**) Instead of ATRA, preincubation of LP-1 cells was done with IFNα for 3 days. These different conditions are analyzed after 4 h of co-cultures between target and effector cells, with and without daratumumab, in different E/T ratios (1:1, 0.5:1 or 0.25:1). All data are representative of eight (*n* = 8) independent experiments and represented as mean ± standard error. Dara: daratumumab. *: *p* < 0.05. **: *p* < 0.01. ***: *p* < 0.001. ****: *p* < 0.0001.

Finally, we tested a combined approach by adding adjuvants to target cells and the blocking nanobody (Nb551) to effector cells. Addition of adjuvants or the blocking nanobody alone did not increase the cytotoxicity towards LP-1 cells. Neither the combination of Nb551 and ATRA (Figure 8A) or Nb551/IFNα (Figure 8B) increased the ADCC results.

In contrast, we observed a major improvement in ADCC towards RPMI-8226 cells after pre-incubation with ATRA/Nb551 (Figure 8C) and IFNα/Nb551 (Figure 8D). Indeed, the percentages of cell lysis increased up to 37% and 18% when Nb551 was combined with ATRA or IFNα respectively. The different strategies tested for RPMI-8226 cells appear to act synergistically to further enhance ADCC lysis.

### 3.6. Inhibition of Inhibitor Receptor NKG2A on NK-92 CD16a Cells with a mAb (CD159a)

**Figure 9:**
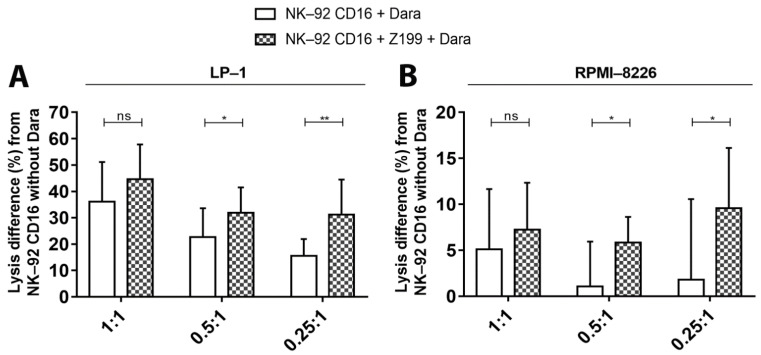
The effect of blocking of NKG2A inhibitory receptors on NK-92—dependent tumor cell cytotoxicity. The differences in LP-1 cell lysis (**A**), RPMI-8226 (**B**) by NK-92 CD16a cells pre-incubated with CD159a during 4 h of co-cultures with or without daratumumab and in different E/T ratios (1:1, 0.5:1 or 0.25:1). All data are representative of at least 3 independent experiments and represented as mean ± standard error. Dara: daratumumab. ns: non-significant. *: *p* < 0.05. **: *p* < 0.01.

NK cells activation is tightly regulated by stimulatory and inhibiting receptors present on their surface. NK-92 cells only have 3 inhibitory receptors on their surface (ILT-2, KIR2DL4 and NKG2A). Whereas blocking ILT-2 does not affect cell lysis of MM cells [25], the implication of the other two receptors in NK cell activity against MM has not been studied. KIR2DL4 is the only KIR receptor expressed on the surface of NK-92 cells. Its ligand, HLA-G, could not be detected on the surface of our MM cell lines. HLA-E, the ligand of NKG2A receptor, could be detected and allowed us to study the cytotoxic activity of NK-92 CD16a effector cells after blocking this NKG2A receptor with a NKG2A-neutralizing mAb. Co-culture with LP-1 cells resulted in an increase of daratumumab-mediated cytotoxicity up to 16% compared to controls (Figure 9A).

Similarly, modest, but significant increases were observed in ADCC towards RPMI-8226 cells (Figure 9B). Indeed, we were able to see increases of 2.1%, 4.8% and 7.8% for the three E/T ratios, respectively, of cytotoxicity of NK-92 CD16a cells pre-incubated with the blocking mAb towards RPMI-8226 cells in presence of daratumumab. Combining the neutralizing mAb with ATRA or IFNα did not increase the lysis of LP-1 or RPMI-8226 (Figure S6).

## 4. Discussion

It was previously demonstrated that CD38 expression levels correlate with the sensitivity of both primary MM cells and cell lines to daratumumab [24]. Our study stressed that the balance between different levels of CD38 expression on target cells and effector cells is essential for the ADCC mechanism towards MM cell lines. Using four MM cell lines with different levels of CD38 expression (LP-1 > MOLP-2 > U266 > RPMI-8226) allowed us to observe ADCC results that correlated with their expression levels (Figure 3C,D and Figure 4A,B). In fact, higher CD38 expression was associated with higher percentages of daratumumab-mediated cell lysis by ADCC, and vice versa.

However, CD38 expression on effector cells must be taken into account, because primary NK-cells and NK-92 CD16a also express CD38 resulting in a fratricide phenomenon following addition of daratumumab [14,15]. Percentages of fratricide when NK-92 CD16a cells were co-cultured with different MM cell lines also varied according to levels of CD38 expression. Indeed, if CD38-expression is higher on target cells compared to effector cells, the balance between ADCC and fratricide leans towards lysis of target cells. In contrast, the balance between ADCC and fratricide leans towards effector cell lysis when the latter express more CD38 than target cells.

In co-cultures with LP-1, daratumumab induced low percentages of fratricide after 2 h and 4 h of incubation. In contrast, strong increases in ADCC were observed. After 2 h of co-culture, ADCC is 2 to 3 times higher than fratricide and 6 times higher after 4 h of incubation. The balance will be reversed after 18 h of incubation because many LP-1 cells have been lysed and thus NK-92 CD16a effector cells will start killing each other resulting in phenomenon of fratricide. For RPMI-8226 cells, we were able to observe, on the contrary, that the balance leans more towards fratricide between effector cells (Figure 4D). This could be easily explained by the lower number of CD38 molecules on the surface of RPMI-8226 cells compared to NK-92 CD16a cells. Indeed, fratricide is up to 10 times higher after 4 h of incubation. The different observations of the other two MM cell lines expressing intermediate CD38 expression levels (U266 and MOLP-2), allowed us to confirm the relation between ADCC, fratricide and CD38 density on the surface of target cells and effector cells (Figure 5B). This equilibrium can be adapted by various strategies that change CD38 expression on target and effector cells.

One way to impact this balance is to prevent the phenomenon of fratricide. Different groups have already tested different strategies in order to overcome this fratricide problem. Kararoudi et al. used the CRISPR-Cas9 system in order to suppress CD38 and thus generate CD38-KO NK cells [26]. Sarkar et al. generated CD38low NK cells who transiently express CD16a after mRNA electroporation. These genetic changes bypass the problem of fratricide while achieving high levels of CD16a receptor expression [27]. In our studies, we used a nanobody binding to the same epitope as daratumumab. Pre-incubating the NK-92 CD16a cells with this nanobody prevents the binding of daratumumab to effector cells and the fratricide (Figure 7). The absence of gene editing is one of the advantages of this technique. It allows to work with NK-cell lines or primary cells that do not require genetic manipulating, which greatly simplifies handling and reduces costs.

Increasing the CD38 expression on target cells is another way to modify the ADCC/fratricide balance in favor of the ADCC. Studies have already tried to improve responses mediated by daratumumab by increasing CD38 expression thanks to all-trans retinoic acid (ATRA) since the latter directly controls the CD38 transcription [24,28,29]. ATRA upregulated CD38 expression in cell lines tested and in primary MM cells, and increased daratumumab-mediated lysis in ADCC assays [24]. This strategy is currently being investigated in a clinical trial combining daratumumab with ATRA in patients with MM (ClinicalTrials.gov Identifier: NCT02751255).

In our study, we were able to confirm this observation on other MM cell lines, U266 and MOLP-2 cell lines, which had not yet been tested (Figure 6D). Another recently published strategy is inhibition of histone deacetylase 6 (HDAC6). A new inhibitor, ricolinostat, up-regulated CD38 on the surface of MM cells and was able to increase cell lysis when combined with daratumumab [30]. IFNα has been previously evaluated in T-cell leukemia. This adjuvant increased CD38 density on the surface of these leukemia cells [29]. In our study, we were able to demonstrate that IFNα increases CD38 expression on the surface of MM cells, but to a lesser extent compared to ATRA (Figure 6A). Takeda company has started a phase I clinical study (ClinicalTrials.gov Identifier: NCT03215030) in which they are evaluating TAK-573 which is a fusion between two attenuated alpha-2b interferon molecules (IFNα2b) and an IgG4 anti-CD38 monoclonal antibody. This construction makes it possible to considerably reduce potential for off-target toxicity thanks to its specificity for CD38 and reduced binding affinity of the attenuated IFNα molecules. Non-clinical studies have shown that TAK-573 has robust anti-tumor activity, including complete responses, in MM xenograft models [31]. Thus, different strategies for increasing CD38 expression on the surface of target cells have improved cytotoxic potential of effector cells in presence of daratumumab.

NK-92 cell line derived from a patient with Hodgkin lymphoma and must be irradiated before being infused into a patient to stop their proliferation and thus prevent development of lymphoma. The aim of irradiating effector cells is to stop their proliferation while retaining their cytotoxic activity. Our results indicate that an irradiation dose of 10 Gy is sufficient to stop their proliferation while maintaining the cytotoxic activity of NK-92 CD16a cells towards our MM cell lines (Figure S2). Clinical studies have already been carried out by injecting irradiated NK-92 cells and anti-tumor effects could be observed with no persistent cells after treatment [18,19]. Combining adoptive NK cell therapy with daratumumab is an interesting approach to circumvent the acquired immune suppression in MM patients. The presence of CD16a, the low-affinity receptor for IgG1 and IgG3, on the surface of NK-cells is crucial for ADCC-dependent cell killing by tumor-targeting mAbs [32]. Depending on the expansion and culture conditions, this CD16a expression can decrease, but the combined use of expanded NK-cells (from umbilical cord blood or peripheral blood) with daratumumab shows encouraging results in preclinical studies [33,34]. To avoid daratumumab-mediated fratricide, the CD38-gene can be deleted in expanded primary NK cells without affecting their in vivo persistence or the ADCC activity against MM cell lines and primary MM cells [26]. Several clinical trials, based on the use of autologous or allogeneic NK cells, are being performed in field of multiple myeloma and some of them combine NK-cells with anti-CD38 mAbs (ClinicalTrials.gov Identifier: NCT04558931). Interestingly, NCT04614636 is testing a cellular therapy with induced pluripotent stem cells (iPSC)-derived NK cells who were further engineered to express a novel high-affinity CD16a receptor, an IL-15 receptor and a deletion of CD38.

Finally, blocking the inhibitory receptor NKG2A on effector cells increased their cytotoxicity towards MM cells, however moderately (Figure 9). Thus, ADCC is largely influenced by CD38 expression levels on the surface of target cells and effector cells, but other variables (such as the presence of inhibitory receptors on the surface of NK cells) also impact cytotoxic effects.

Although monotherapy with daratumumab causes a rapid depletion of NK cells [14,15], the clinical efficacy of this mAb is not affected by this decrease. Residual NK cells, with low levels of CD38-expression, retain their cytotoxic function and thus are still capable of ADCC [14,15]. These residual cells, possessing a CD38-/low phenotype, have been demonstrated to be highly proliferative and more efficient in eradicating MM cells than CD38+ NK cells. The use of NK cells weakly expressing CD38 could thus be a strategy to optimize the efficacy of daratumumab [16]. ADCC and effector cell death were also assessed in our cytotoxicity assays using primary NKs as effector cells (Figure S7). In addition to having significantly lower levels of total NK cells, patients treated with daratumumab show an increase in the activated CD38−/low NK cell population (CD69+), associated with an increase in activation of CD8+ T cells [35]. These two daratumumab-related modifications support an adaptive response in patients which may contribute to the depth of response seen in patients treated with daratumumab [9,10,12,13].

In addition to its direct anti-tumor effects, daratumumab also plays an immunomodulatory role. Indeed, they induce the expansion of cytotoxic T lymphocytes; reduction of immunosuppressive cells, including suppressor cells derived from CD38+ myeloid, CD38+ regulatory B cells and a CD38+ regulatory T subpopulation (CD4+ CD25+ CD127dim), to promote T cell activity against MM cells; and increased T cell repertoire clonality, reduction of NK cells, and downregulation of CD38 on target cells [14,36]. This results in prolonged and profound clinical responses in MM patients treated with daratumumab. Further investigations should be carried out to verify the immunomodulatory role of daratumumab via cells of the NK-92 lineage.

In conclusion, by using NK-92 cell line, which is off-the-shelf available, we showed that the balance between CD38 levels on effector and target cells is crucial for induction of ADCC. This cytotoxicity could be further increased by either upregulating CD38 expression or neutralizing CD38 on effector cells with a blocking nanobody. NK-92 cells, armed with CD16a receptor, are a valuable source of effector cells to improve antibody-mediated cytotoxicity.

## Data Availability

Not applicable.

## Supplementary Materials

The following are available online at https://www.mdpi.com/article/10.3390/cancers13123072/s1, Figure S1: Gating strategy for NK-92—dependent tumor cell cytotoxicity. Figure S2: NK-92—dependent tumor cell cytotoxicity. Figure S3: Human multiple myeloma cell lines, including LP-1 and RPMI-8226 cells, overexpressed CD38. Figure S4: The effect of increasing CD38 density on NK-92—dependent tumor cell cytotoxicity, measured by calcein release assay. Figure S5: The effect of a blocking anti-CD38 nanobody (Nb551) on NK-92—dependent tumor cell cytotoxicity measured by calcein release assay. Figure S6: The effect of combining NKG2A blocking antibodies with CD38-upregulation on NK-92—dependent tumor cell cytotoxicity. Figure S7: Primary NK—dependent tumor cell cytotoxicity and fratricide.

## References

[1] Cho S.-F., Lin L., Xing L., Yu T., Wen K., Anderson K.C., Tai Y.-T. (2017). Monoclonal Antibody: A New Treatment Strategy against Multiple Myeloma. Antibodies.

[2] Abramson H.N. (2018). Monoclonal Antibodies for the Treatment of Multiple Myeloma: An Update. Int. J. Mol. Sci..

[3] Fast L.D., Hansen J.A., Newman W. (1981). Evidence for T cell nature and heterogeneity within natural killer (NK) and antibody-dependent cellular cytotoxicity (ADCC) effectors: A comparison with cytolytic T lymphocytes (CTL). J. Immunol..

[4] Mahaweni N.M., Bos G.M.J., Mitsiades C.S., Tilanus M.G.J., Wieten L. (2018). Daratumumab augments alloreactive natural killer cell cytotoxicity towards CD38+ multiple myeloma cell lines in a biochemical context mimicking tumour microenvironment conditions. Cancer Immunol. Immunother..

[5] Laubach J.P., Tai Y.-T., Richardson P.G., Anderson K.C. (2014). Daratumumab granted breakthrough drug status. Expert Opin. Investig. Drugs.

[6] Deaglio S., Mehta K., Malavasi F. (2001). Human CD38: A (r)evolutionary story of enzymes and receptors. Leuk. Res..

[7] Wong S.W., Comenzo R.L. (2015). CD38 Monoclonal Antibody Therapies for Multiple Myeloma. Clin. Lymphoma Myeloma Leuk..

[8] McKeage K. (2016). Daratumumab: First Global Approval. Drugs.

[9] Lonial S., Weiss B.M., Usmani S.Z., Singhal S., Chari A., Bahlis N.J., Belch A., Krishnan A., Vescio R.A., Mateos M.V. (2016). Daratumumab monotherapy in patients with treatment-refractory multiple myeloma (SIRIUS): An open-label, randomised, phase 2 trial. Lancet.

[10] Lokhorst H.M., Plesner T., Laubach J.P., Nahi H., Gimsing P., Hansson M., Minnema M.C., Lassen U., Krejcik J., Palumbo A. (2015). Targeting CD38 with Daratumumab Monotherapy in Multiple Myeloma. N. Engl. J. Med..

[11] Bahlis N.J., Dimopoulos M.A., White D.J., Benboubker L., Cook G., Leiba M., Ho P.J., Kim K., Takezako N., Moreau P. (2020). Daratumumab plus lenalidomide and dexamethasone in relapsed/refractory multiple myeloma: Extended follow-up of POLLUX, a randomized, open-label, phase 3 study. Leukemia.

[12] Palumbo A., Chanan-Khan A., Weisel K., Nooka A.K., Masszi T., Beksac M., Spicka I., Hungria V., Munder M., Mateos M.V. (2016). Daratumumab, Bortezomib, and Dexamethasone for Multiple Myeloma. N. Engl. J. Med..

[13] Dimopoulos M., Oriol A., Nahi H., San-Miguel J., Bahlis N.J., Usmani S.Z., Rabin N., Orlowski R., Komarnicki M., Suzuki K. (2016). Daratumumab, Lenalidomide, and Dexamethasone for Multiple Myeloma. N. Engl. J. Med..

[14] Nijhof I.S., Casneuf T., Van Velzen J., Van Kessel B., Axel A.E., Syed K., Groen R.W.J., Van Duin M., Sonneveld P., Minnema M.C. (2016). CD38 expression and complement inhibitors affect response and resistance to daratumumab therapy in myeloma. Blood.

[15] Casneuf T., Xu X.S., Adams H.C., Axel A.E., Chiu C., Khan I., Ahmadi T., Yan X., Lonial S., Plesner T. (2017). Effects of daratumumab on natural killer cells and impact on clinical outcomes in relapsed or refractory multiple myeloma. Blood Adv..

[16] Wang Y., Zhang Y., Hughes T., Zhang J., Caligiuri M.A., Benson D.M., Yu J. (2018). Fratricide of NK Cells in Daratumumab Therapy for Multiple Myeloma Overcome by Ex Vivo–Expanded Autologous NK Cells. Clin. Cancer Res..

[17] Tonn T., Schwabe D., Klingemann H.G., Becker S., Esser R., Koehl U., Suttorp M., Seifried E., Ottmann O., Bug G. (2013). Treatment of patients with advanced cancer with the natural killer cell line NK-92. Cytotherapy.

[18] Arai S., Meagher R., Swearingen M., Myint H., Rich E., Martinson J., Klingemann H. (2008). Infusion of the allogeneic cell line NK-92 in patients with advanced renal cell cancer or melanoma: A phase I trial. Cytotherapy.

[19] Williams B.A., Law A.D., Routy B., Denhollander N., Gupta V., Wang X.-H., Chaboureau A., Viswanathan S., Keating A. (2017). A phase I trial of NK-92 cells for refractory hematological malignancies relapsing after autologous hematopoietic cell transplantation shows safety and evidence of efficacy. Oncotarget.

[20] Clémenceau B., Vivien R., Pellat C., Foss M., Thibault G., Vié H. (2013). The human natural killer cytotoxic cell line NK-92, once armed with a murine CD16 receptor, represents a convenient cellular tool for the screening of mouse mAbs according to their ADCC potential. MAbs.

[21] Slaymaker I.M., Gao L., Zetsche B., Scott D.A., Yan W.X., Zhang F. (2016). Rationally engineered Cas9 nucleases with improved specificity. Science.

[22] Emi N., Friedmann T., Yee J.K. (1991). Pseudotype formation of murine leukemia virus with the G protein of vesicular stomatitis virus. J. Virol..

[23] Li T., Qi S., Unger M., Hou Y.N., Deng Q.W., Liu J., Lam C.M.C., Wang X.W., Xin D., Zhang P. (2016). Immuno-targeting the multifunctional CD38 using nanobody. Sci. Rep..

[24] Nijhof I.S., Groen R.W.J., Lokhorst H.M., Van Kessel B., Bloem A.C., Van Velzen J., De Jong-Korlaar R., Yuan H., Noort W.A., Klein S.K. (2015). Upregulation of CD38 expression on multiple myeloma cells by all-trans retinoic acid improves the efficacy of daratumumab. Leukemia.

[25] Heidenreich S., Eulenburg C.Z., Hildebrandt Y., Stubig T., Sierich H., Badbaran A., Eiermann T.H., Binder T.M.C., Kroger N. (2012). Impact of the NK cell receptor LIR-1 (ILT-2/CD85j/LILRB1) on cytotoxicity against multiple myeloma. Clin. Dev. Immunol..

[26] Kararoudi M.N., Nagai Y., Elmas E., Pereira M.D.S.F., Ali S.A., Imus P.H., Wethington D., Borrello I.M., Lee D.A., Ghiaur G. (2020). CD38 deletion of human primary NK cells eliminates daratumumab-induced fratricide and boosts their effector activity. Blood.

[27] Sarkar S., Chauhan S.K.S., Daly J., Natoni A., Fairfield H., Henderson R., Nolan E., Swan D., Hu J., Reagan M.R. (2020). The CD38low natural killer cell line KHYG1 transiently expressing CD16F158V in combination with daratumumab targets multiple myeloma cells with minimal effector NK cell fratricide. Cancer Immunol. Immunother..

[28] Lewandowski D., Linassier C., Iochmann S., Degenne M., Domenech J., Colombat P., Binet C., Herault O. (2002). Phosphatidylinositol 3-kinases are involved in the all-trans retinoic acid-induced upregulation of CD38 antigen on human haematopoietic cells. Br. J. Haematol..

[29] Mihara K., Yoshida T., Ishida S., Takei Y., Kitanaka A., Shimoda K., Morishita K., Takihara Y., Ichinohe T. (2016). All-trans retinoic acid and interferon-α increase CD38 expression on adult T-cell leukemia cells and sensitize them to T cells bearing anti-CD38 chimeric antigen receptors. Blood Cancer J..

[30] García-Guerrero E., Götz R., Doose S., Sauer M., Rodríguez-Gil A., Nerreter T., Kortüm K.M., Pérez-Simón J.A., Einsele H., Hudecek M. (2021). Upregulation of CD38 expression on multiple myeloma cells by novel HDAC6 inhibitors is a class effect and augments the efficacy of daratumumab. Leukemia.

[31] Fatholahi M., Valencia M., Mark A., Bi M., Syed S., Zhang Y., Taura T., Yun Y., Wilson D., Chattopadhyay N. (2019). TAK-573, an anti-CD38-targeted attenuated interferon alpha (IFNα) fusion protein, showed anti-myeloma tumor responses in combination with standard of care (SOC) agents in multiple myeloma (MM) xenograft tumor models in vivo. Clin. Lymphoma Myeloma Leuk..

[32] Capuano C., Pighi C., Battella S., De Federicis D., Galandrini R., Palmieri G. (2021). Harnessing CD16-Mediated NK Cell Functions to Enhance Therapeutic Efficacy of Tumor-Targeting mAbs. Cancers.

[33] Motais B., Charvátová S., Walek Z., Hrdinka M., Smolarczyk R., Cichoń T., Czapla J., Giebel S., Šimíček M., Jelínek T. (2021). Selection, Expansion, and Unique Pretreatment of Allogeneic Human Natural Killer Cells with Anti-CD38 Monoclonal Antibody for Efficient Multiple Myeloma Treatment. Cells.

[34] Reina-Ortiz C., Constantinides M., Fayd-Herbe-De-Maudave A., Présumey J., Hernandez J., Cartron G., Giraldos D., Díez R., Izquierdo I., Azaceta G. (2020). Expanded NK cells from umbilical cord blood and adult peripheral blood combined with daratumumab are effective against tumor cells from multiple myeloma patients. OncoImmunology.

[35] Adams H.C., Stevenaert F., Krejcik J., van der Borght K., Smets T., Bald J., Abraham Y., Ceulemans H., Chiu C., Vanhoof G. (2019). High-Parameter Mass Cytometry Evaluation of Relapsed/Refractory Multiple Myeloma Patients Treated with Daratumumab Demonstrates Immune Modulation as a Novel Mechanism of Action. Cytometry A.

[36] Krejcik J., Casneuf T., Nijhof I.S., Verbist B., Bald J., Plesner T., Syed K., Liu K., Van De Donk N.W.C.J., Weiss B.M. (2016). Daratumumab depletes CD38+ immune regulatory cells, promotes T-cell expansion, and skews T-cell repertoire in multiple myeloma. Blood.

