# Supplementary material for: Balancing the CD38 Expression on Effector and Target Cells in Daratumumab-Mediated NK Cell ADCC against Multiple Myeloma
Source: Cancers (Basel). 2021 Jun 20;13(12):3072. doi: 10.3390/cancers13123072 (PMC8234557; doi:10.3390/cancers13123072)
Supplement: Supplementary file 1 [file cancers-13-03072-s001.zip › cancers-1189173-supplementary.pdf]

Supplementary Materials

Balancing the CD38 Expression on Effector and Target Cells in Daratumumab-Mediated NK Cell ADCC against Multiple Myeloma

Margaux Lejeune [1], Elodie Duray [1], Matthias Peipp [2], Béatrice Clémenceau [3], Frédéric Baron [1,4], Yves Beguin [1,4] and Jo Caers [1,4,*]

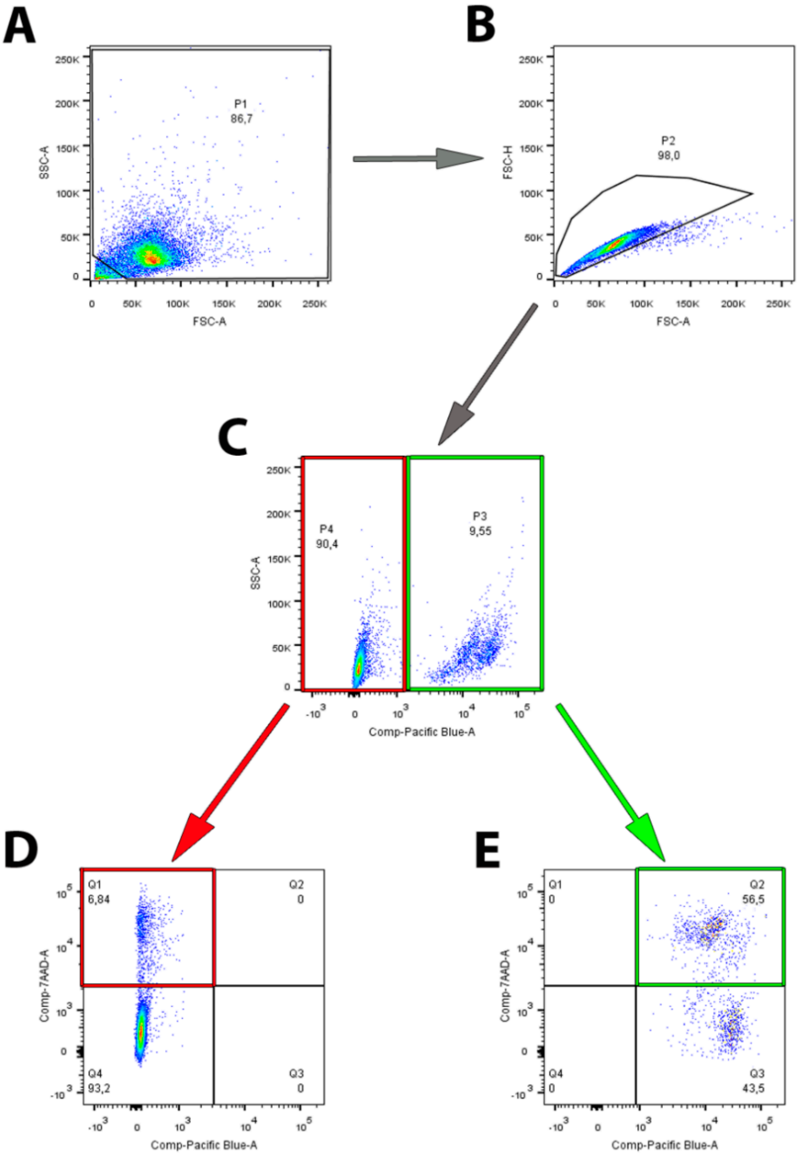

Figure S1. Gating strategy for NK-92 – dependent tumor cell cytotoxicity measured by 6 hours flow cytometry. **(A)** The entire cell population is taken into account on the FSC-SSC plots, except for the debris at the bottom left, and **(B)** single cells ('singlets') are retained. **(C)** Cells are then discriminated into target cells (green) and effector cells (red) thanks to violet dye which is positive only for target cells. **(D)** We then take into account percentage of dead cells (7-AAD +) in negative-violet dye population to assess fratricide. **(E)** In parallel, we assess percentage of dead cells (7-AAD +) positive-violet dye population to assess cytotoxicity.

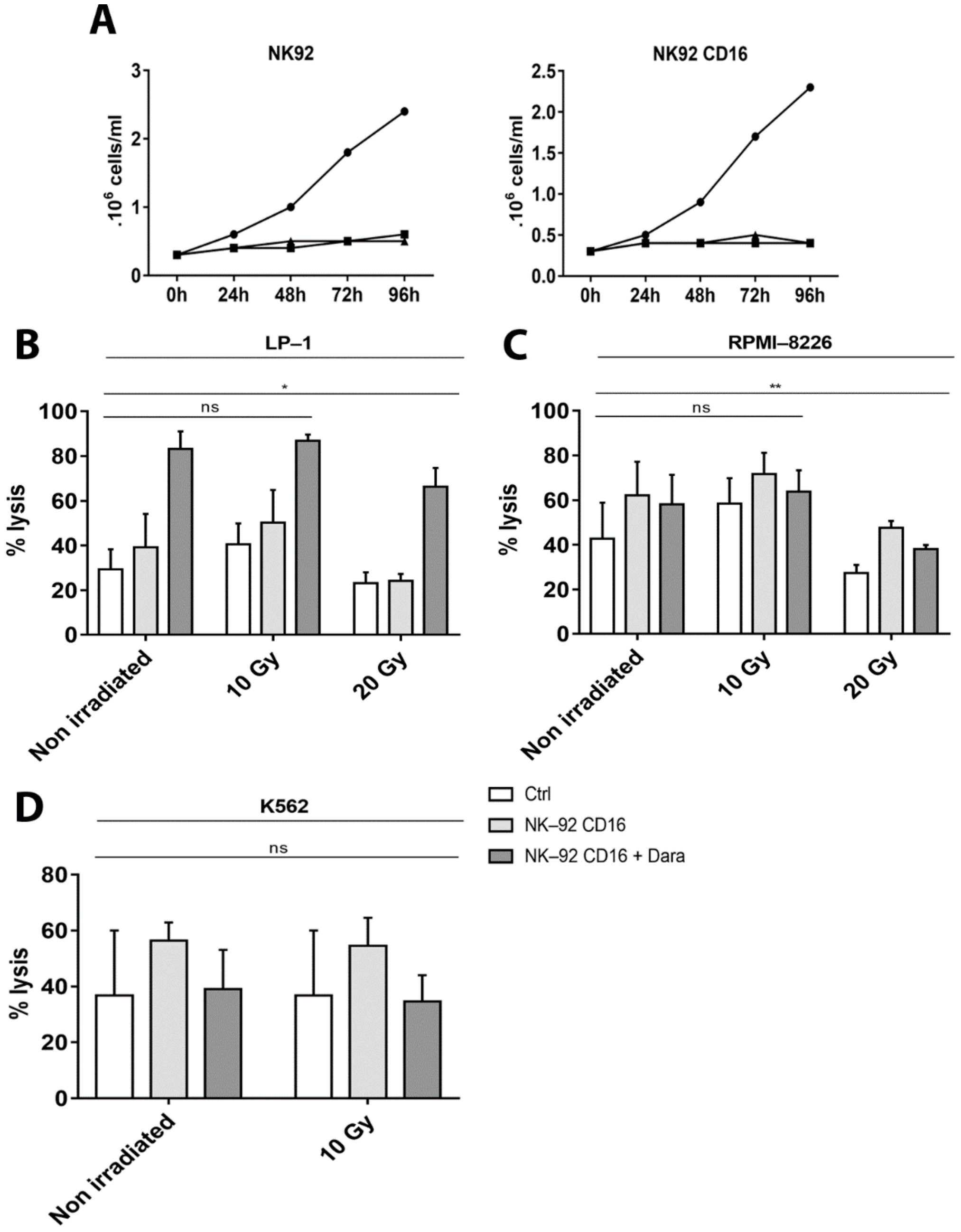

Figure S2. NK-92 – dependent tumor cell cytotoxicity measured by 6 hours flow cytometry-based assay with CD38-expressing target cells. **(A)** Effect of irradiation on the proliferation of NK-92 and NK-92 CD16 cells. Lysis percentages of LP-1 **(B)**, RPMI-8226 **(C)** and K562 **(D)** cells after 6 hours of incubation and in the presence of non-irradiated or irradiated with 10 or 20 Gy NK-92 CD16 cells (effector/target cells=10:1). The following conditions were tested: with or without (NK-92 CD16) or NK-92 CD16 combined with daratumumab. There is no significant difference between ADCC results without prior irradiation or after 10 Gy irradiation of NK-92 CD16 cells for LP-1 cell line **(B)**, RPMI-8226 cell line **(C)**, or negative control leukemia cell line K562 **(D)**. On the other hand, we were able to observe a significant decrease in percentages of ADCC following irradiation of 20 Gy of NK-92 CD16 cells. All data are representative of fifteen (for non-irradiated cells) and four (for irradiated cells) (n = 15 or 4) independent experiments and represented as mean +/− standard error. ns: non-significant. *: $p < 0.05$. **: $p < 0.01$.

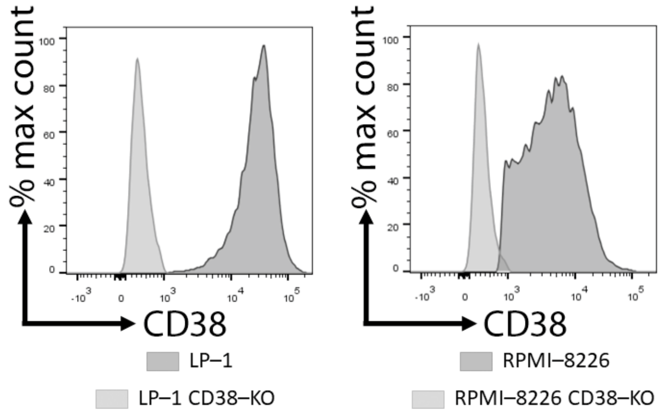

Figure S3. Human multiple myeloma cell lines, including LP-1 and RPMI-8226 cells, overexpressed CD38. We inactivated CD38 gene by using the CRISPR/Cas9 technology, to obtain CD38KO cell lines. CD38 expression was assessed by flow cytometry. Results are representative of three independent experiments (n = 3). We confirmed that parental cell lines present varying levels of CD38 expression while CD38-KO cell lines no longer express CD38.

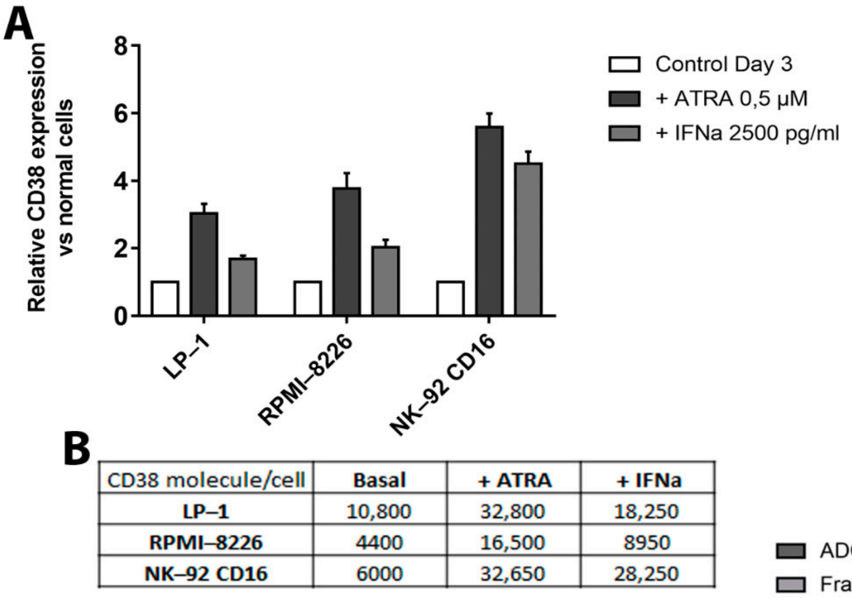

B

CD38 molecule/cell	Basal	+ ATRA	+ IFNa
**LP–1**	10,800	32,800	18,250
**RPMI–8226**	4400	16,500	8950
**NK–92 CD16**	6000	32,650	28,250

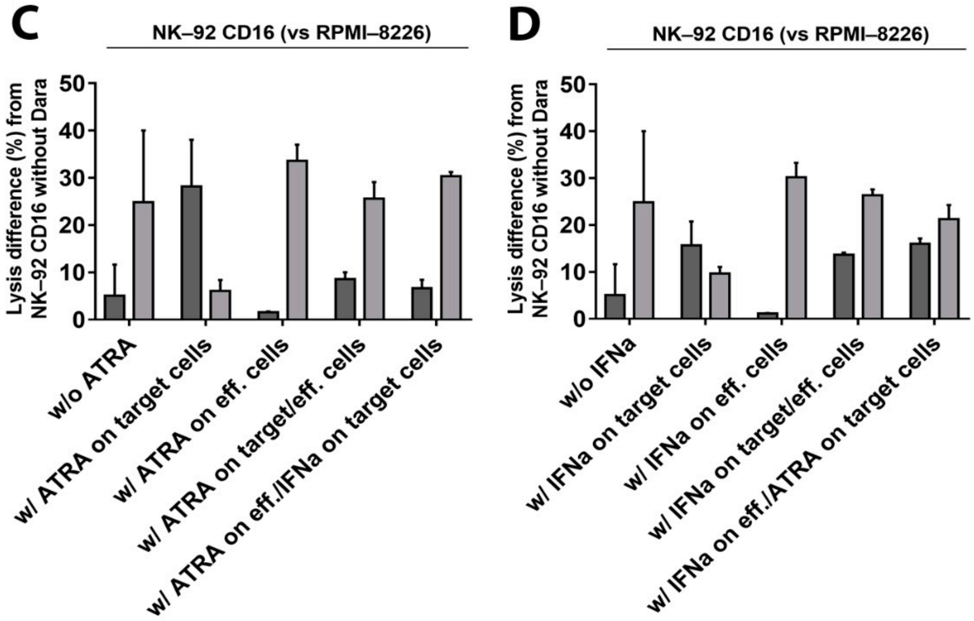

Figure S4. The effect of increasing CD38 density on NK-92 – dependent tumor cell cytotoxicity, measured by calcein release assay. **(A)** Relative expressions of CD38 and **(B)** quantification of the number of CD38 molecules per cell after 3 days incubation of LP-1, RPMI-8226 and NK-92 CD16 with 0.5 μM of ATRA or with 2500 pg/ml of IFNα compared to cells alone. **(C)** Preincubation of target or effector cells with ATRA and the effects on ADCC (dark grey) and fratricide (light grey). Each graph shows the results obtained without pre-incubation with adjuvants (w/o ATRA or IFNa), after pre-incubation of target cells with adjuvants (w/ ATRA or IFNa on target cells), after pre-incubation of effector cells with adjuvants (w/ ATRA or IFNa on eff. cells) or after pre-incubation of target and effector cells with adjuvants (w/ ATRA or IFNa on target cells). These results were observed after 4 hours of incubation and at effector to target cells ratio of 1:1. All data are representative of at least 3 independent experiments and represented as mean +/− standard error.

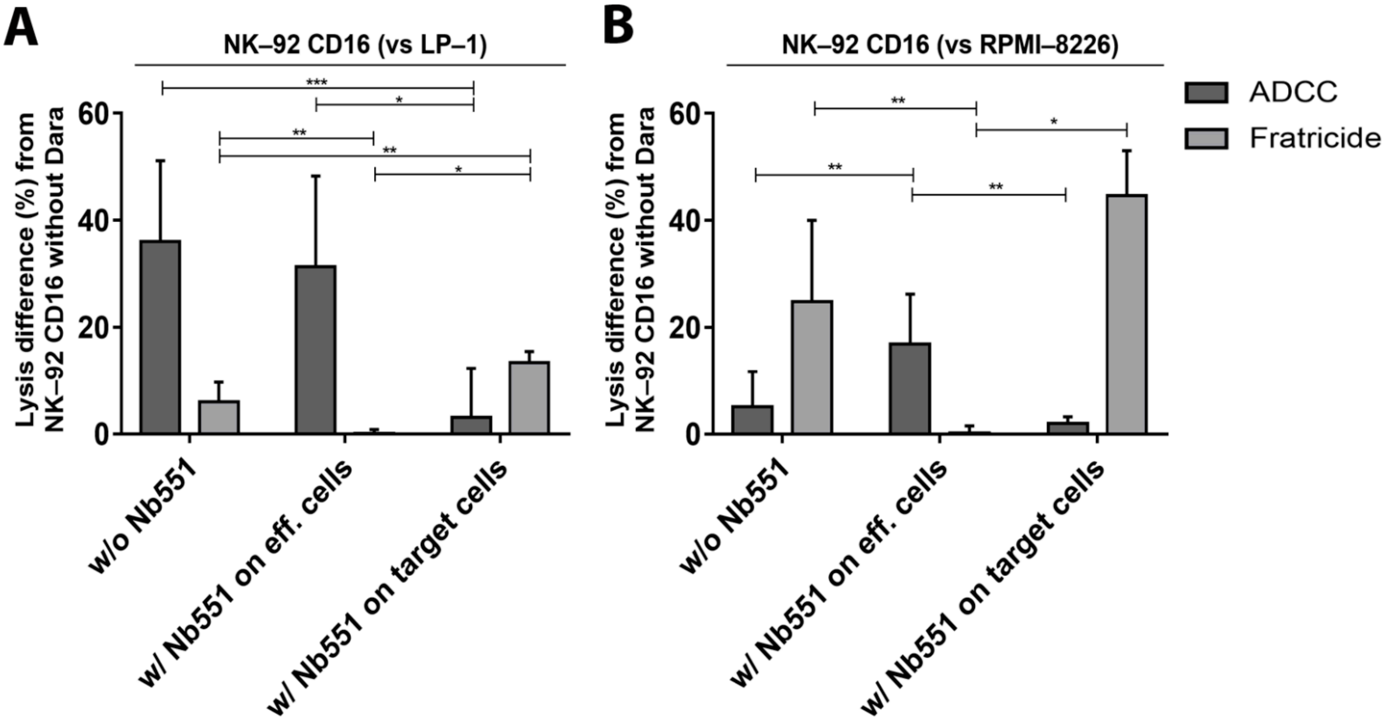

Figure S5. The effect of a blocking anti-CD38 nanobody (Nb551) on NK-92 – dependent tumor cell cytotoxicity measured by calcein release assay. Target-cell lysis (ADCC) and effector-cell lysis (Fratricide) in LP-1 **(A)** or RPMI-8226 **(B)** and NK-92 CD16 co-cultures without the presence of Nb551 (w/o Nb551), after pre-incubation of effector cells with Nb551 (w/ Nb551 on eff. cells) or after pre-incubation of target cells with Nb551 (w/ Nb551 on target cells). These results were observed after 4 hours of incubation and at effector to target cells ratio of 1:1. All data are representative of at least 4 independent experiments and represented as mean +/− standard error. *: $p < 0.05$. **: $p < 0.01$. ***: $p < 0.001$.

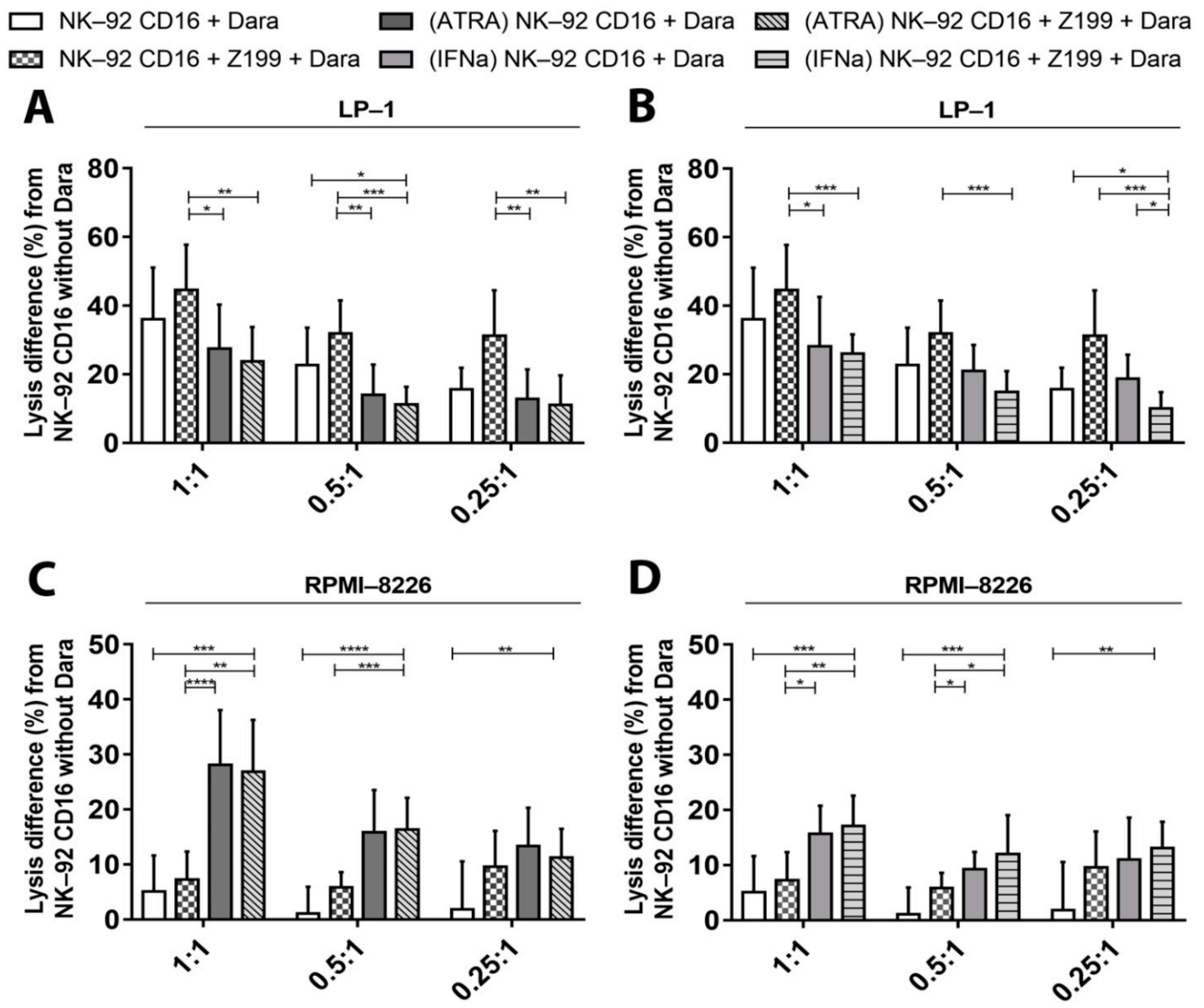

Figure S6. The effect of combining NKG2A blocking antibodies with CD38-upregulation on NK-92 – dependent tumor cell cytotoxicity, quantified by calcein release assay **(A)** Lysis differences in percentage of LP-1, pre-incubated or not with ATRA for 3 days, by NK-92 CD16 cells pre-incubated or not with anti-NKG2A mAb Z199 or ATRA + Z199 combination. **(B)** Lysis differences in percentage of LP-1, pre-incubated or not with IFNα for 3 days, by NK-92 CD16 cells pre-incubated or not with Z199, or IFNα + Z199combination. **(C)** Lysis differences in percentage of RPMI-8226, pre-incubated or not with ATRA for 3 days, by NK-92 CD16 cells pre-incubated or not with Z199, or ATRA + Z199combination. **(D)** Lysis differences in percentage of RPMI-8226, pre-incubated or not with IFNα for 3 days, by NK-92 CD16 cells pre-incubated or not with Z199, or IFNα + Z199combination. These different conditions were analyzed after 4 hours of incubation between conditions in presence of NK-92 CD16 cells without daratumumab and corresponding conditions in presence of daratumumab, in different E/T ratios (1:1, 0.5:1 or 0.25:1). All data are representative of seven (n = 7) independent experiments and represented as mean +/− standard error. Dara: daratumumab. *: $p < 0.05$. **: $p < 0.01$. ***: $p < 0.001$. ****: $p < 0.0001$.

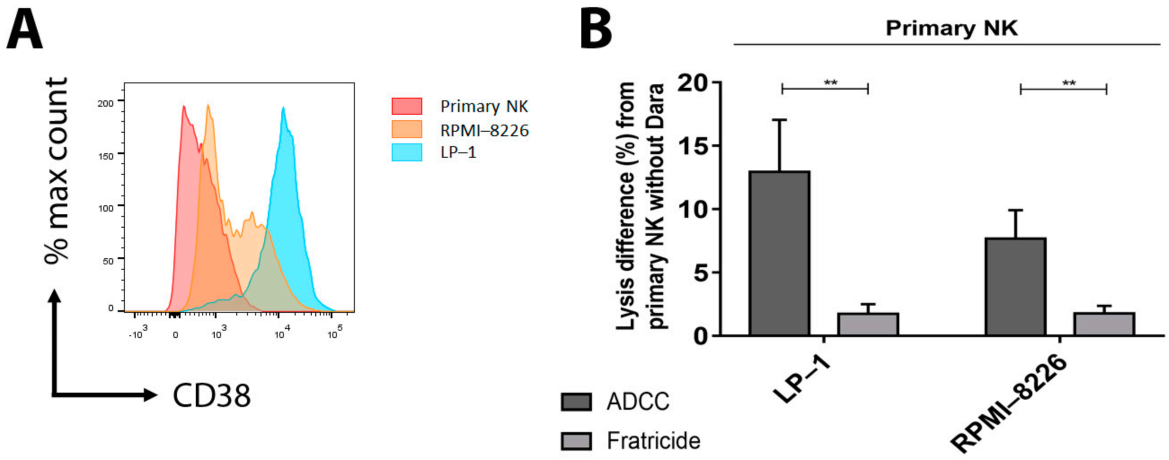

Figure S7. Primary NK – dependent tumor cell cytotoxicity and fratricide measured by calcein release assay. **(A)** CD38 expression in established cell lines (LP-1 and RPMI-8226) and in primary NK from buffy coat. Graph is representative of three independent experiments (n = 3). **(B)** Comparison of lysis differences in percentages of ADCC and fratricide in co-cultures with LP-1/Primary NK or RPMI-8226/Primary NK co-cultures after 4 of incubation and with E/T ratio of 1:1. All data are representative of six independent experiments and represented as mean +/− standard error. **: $p < 0.01$.
